# Refractory ventricular fibrillations after surgical repair of atrial septal defects in a patient with CACNA1C gene mutation - case report

**DOI:** 10.1186/s13019-017-0683-4

**Published:** 2017-12-19

**Authors:** Ai Kojima, Fumiaki Shikata, Toru Okamura, Takashi Higaki, Seiko Ohno, Minoru Horie, Shunji Uchita, Yujiro Kawanishi, Kenji Namiguchi, Takumi Yasugi, Hironori Izutani

**Affiliations:** 1 Department of Cardiovascular Surgery, Ehime University, Shitsukawa, Toon, Ehime 7910295 Japan; 2 Department of Cardiothoracic Surgery, St Vincent’s Hospital, Sydney, NSW Australia; 3 Department of Pediatric Cardiology, Children’s Medical Center, Ehime University, Ehime, Japan; 4 Department of Cardiovascular and Respiratory Medicine, Shiga University of Medical Science, Shiga, Japan

**Keywords:** Congenital long QT syndrome, Ventricular fibrillation, Adult congenital heart diseases, Atrial septal defect

## Abstract

**Background:**

Congenital long QT syndrome (LQTS) can cause ventricular arrhythmic events with syncope and sudden death resulting from malignant torsades de pointes (TdP) followed by ventricular fibrillations (VFs). However, the syndrome is often overlooked prior to the development of arrhythmic events in patients with congenital heart diseases demonstrating right bundle branch block on electrocardiogram (ECG). We present a case of an adult patient with congenital heart disease who developed VFs postoperatively, potentially due to his mutation in a LQTS related gene, which was not identified on preoperative assessment due to incomplete evaluation of his family history.

**Case presentation:**

A 64-year-old man was diagnosed as having multiple atrial septal defects. He presented with no symptoms of heart failure. His preoperative ECG showed complete right bundle branch block (CRBBB) with a corrected QT interval time of 478 ms. He underwent open-heart surgery to close the defects through median sternotomy access. Three hours after the operation, he developed multiple events of TdP and VFs in the intensive care unit. Cardiopulmonary resuscitation and multiple cardioversions were attempted for his repetitive TdP and VFs. He eventually reverted to sinus rhythm, and intravenous beta-blocker was administered to maintain the sinus rhythm. After this event, his family history was reviewed, and it was confirmed that his daughter and grandson had a medical history of arrhythmia. A genetic test confirmed that he had a missense mutation in CACNA1C, p.K1580 T, which is the cause for type 8.

**Conclusions:**

This case highlights the importance of paying attention to other ECG findings in patients with CRBBB, which can mask prolonged QT intervals.

## Background

Congenital long QT syndrome (LQTS) causes arrhythmic events such as syncope and sudden death due to malignant torsades de pointes (TdP) followed by ventricular fibrillations (VFs). However, in the absence of arrhythmic events, the syndrome can be overlooked, especially in patients with congenital heart diseases, that demonstrate right bundle branch block (RBBB) on electrocardiograms (ECGs) [1, 2]. In addition, QT intervals in ECGs are known to increase for 2 days after open heart surgeries using cardiopulmonary bypasses as a result of electrolyte imbalance and QT prolonging medications. The prolongation of QT interval can trigger arrhythmic events after operations in patients with genetic disorders, such as long QT-related gene mutations or polymorphisms [1, 3]. If the diagnosis of the syndrome is made before open heart surgery, clinicians can avoid factors contributing to the prolongation of QT intervals and, as a result, may be able to prevent ventricular arrhythmias postoperatively [4]. We present an adult patient with congenital heart disease who developed VFs postoperatively, possibly due to his mutation in a LQTS-related gene, which were not identified preoperatively due to incomplete assessment of his family history.

## Case presentation

**Fig. 1:**
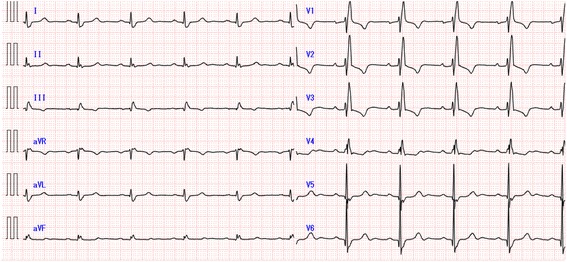
The preoperative electrocardiogram. The preoperative electrocardiogram showed complete right bundle branch block, and the corrected QT interval time was 478 ms

A 64-year-old man with a diagnosis of atrial septal defect (ASD) presented ECG features of incomplete or complete right bundle branch block (CRBBB) during a regular medical check-up. He was asymptomatic, and he had no other significant past medical history. He was referred to our hospital to assess the possibility of catheter device occlusion for ASD, and surgical correction was indicated due to multiple defects and the size of the defects. A chest radiograph showed enlargement of the pulmonary vasculature, indicating pulmonary high flow. An ECG showed CRBBB, and the corrected QT interval (Bazett formula) was 478 ms (Fig. 1). Echocardiography showed preserved left ventricular function, and enlarged right atrium and right ventricle with trivial tricuspid regurgitation and multiple secundum ASDs. The patient was not taking any medications known to prolong the QT interval, and his laboratory data indicated no significant electrolyte disorders. We were informed that he had a child receiving drug treatment for arrhythmia; however, we did not think that this was clinically relevant before the surgery as further information was not provided.

**Fig. 2:**
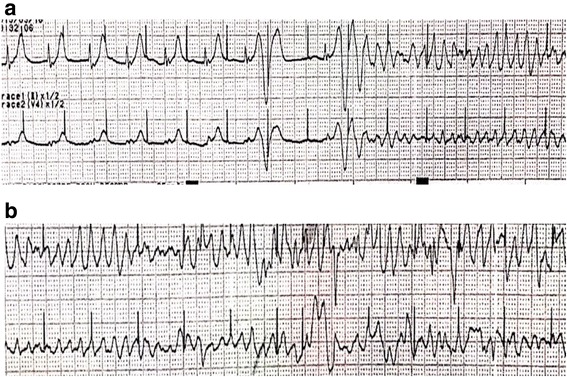
Refractory ventricular fibrillations after the atrial septal defect repair. **a** Three hours after the surgical correction of multiple atrial septal defects, the patient suddenly presented R on T followed by Torsades de Pointes in the intensive care unit, and eventually, the electrocardiogram shifted to a ventricular fibrillation pattern. **b** The patient presented with repetitive ventricular fibrillations even after he reverted to sinus rhythm with direct current cardioversions

During the operation, anesthetic drugs were used as follows: sevoflurane, remifentanil, dexmedetomidine, propofol, fentanil and rocuronium were administered. Cefazolin was given for the prevention of surgical site infection. After the median sternotomy, an aortic cannula was placed in the ascending aorta, and venous cannulas were inserted into the superior vena cava and the inferior vena cava to establish the cardiopulmonary bypass (CPB). Once the aorta was cross-clamped, cold blood cardioplegic solution was infused through the cannula in the ascending aorta. The body temperature was cooled down to 33.8^∘^ Celsius. Under conditions of cardiac arrest, the right atrium was cut horizontally and opened. Then, three large ASDs were identified at the secundum with thin rims around the holes. The ASDs were closed directly with continuous 4–0 Prolene. After the de-airing, the aorta was de-clamped. The cross-clamp time was 38 min. The patient developed VF. The direct current (DC) cardioversion was successful to convert to sinus rhythm with one attempt of 10 J. The CPB was weaned uneventfully, and its running time was 76 min. The chest was closed using standard techniques, and the patient was sent to the intensive care unit (ICU) with tracheal intubation. In the ICU, dopamine at 4 mcg/kg/min and noradrenaline at 0.05 mcg/kg/min were administered for cardiac support. Propofol and dexmedetomidine were given at the adequate level for sedation. Bipolar temporary pacing wire was placed on the right atrium, and the setting of pacemaker was AAI pacing mode with HR 88 bpm, the sensitivity threshold of 3 mV and the output of 10 mA. The serum level of potassium was 3.8 mEq/l (normal serum potassium value: 3.8–4.8 mmol/l), and that of magnesium was not measured 2 h after the surgery as we do not routinely measure it after low risk operations and the patient was stable. Three hours after the operation, he suddenly presented R on T followed by TdP and Vfs (Fig. 2a). Chest compressions were initiated, and DC cardioversion was attempted. Sinus rhythm was achieved following cardioversion; however, the patient presented repetitive VF (14 times) (Fig. 2b).

Intravenous amiodarone and lidocaine were not effective in managing the arrhythmic storm. After the administration of magnesium and nifecalant, the patient finally recovered to sinus rhythm. Echocardiography revealed normal left ventricular wall motion, which indicated no heart ischemia. After the event, he underwent 24 h of hypothermic treatment to prevent brain damage, and, fortunately, there remained no evidence of brain damage on computed tomography. Landiolol had been administered intravenously to maintain sinus rhythm until the patient was able to take bisoprolol tablets orally. Given that his VFs were refractory and the patient had no coronary artery disease and relevant valve disease, we supposed that he might have been a carrier of familial arrhythmic disorders causing repetitive VFs. Therefore, we reviewed his family history and found that his daughter and grandson had congenital LQTS diagnosed at other hospitals, and his grandson was being treated with a beta blocker for this disease.

**Fig. 3:**
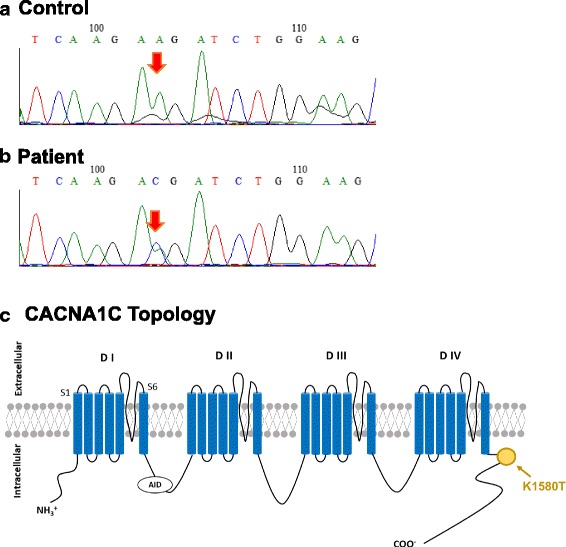
The result of genetic analysis. **a** and **b** A heterozygous nucleotide substitution from (**a** to **c**) (red allows) was shown in the patient (**b**) compared to control (**a**). **c** The topology of K1580 T mutation in CACNA1C gene

After obtaining informed consent from the patient, genetic screening for LQTS related genes was conducted to confirm the diagnosis. In the genetic analysis, a CACNA1c missense mutation, c. 4739a > c, p.K1580 T, was identified. CACNA1C is a causative gene for LQTS type 8 (Fig. 3(a)-(c)) [5–7]. The mutation was novel and not reported in the cohort databases; ExAC (http://exac.broadinstitute.org/), gnomAD (http://gnomad.broadinstitute.org/) and HGVD (http://www.hgvd.genome.med.kyoto-u.ac.jp/index.html?). We could not perform genetic analysis for the family members who were diagnosed with LQTS.

He was discharged 2 weeks after the operation with the prescription of bisoprolol.

## Discussion

Congenital LQTS is a disease resulting from impaired expression or dysfunctions of cardiac ion channels as a result of their encoding gene variants. It can cause the prolongation of QT intervals and, as a consequence, result in life threatening events such as VFs. Itoh et al. reported that 28% of acquired LQTS patients were actually “congenital” [8]. Moreover, only 60% of patients show symptoms prior to the diagnosis of congenital LQTS [9]. Given that our patient was asymptomatic until the operation, it is presumable that stimulating factors related to perioperative and postoperative procedures or the use of medicines strongly contributed to the event of postoperative TdP followed by VFs. The factors associated with fatal arrhythmias for acquired LQTS are medicines including anesthetic agents, inotropes, hypokalemia, bradycardia, emotional stress, hypothermia and the combinations of these factors [8, 10]. If we had known that the patient had a genetic background of CACNA1C mutations, some of which were reported as cardiac-only Timothy syndrome, we could have avoided potential factors that triggered malignant TdP and VFs [5–7, 10]. In order to prevent malignant arrhythmias postoperatively, some authors have recommended the use of appropriate premedications, avoidance of hypothermia, proper anesthetic agents, adequate pain relief, continuous use of beta blockers during and after operations, avoidance of the excess use of diuretics, and maintenance of proper level of electrolytes such as calcium and magnesium [3, 4, 10, 11]. In our case, sevoflurane, propofol, dopamine and dexmedetomidine were used for anesthesia and postoperative care, and these were categorized as possible cause of arrhythmic events or should be avoided in CredibleMeds (https://www.crediblemeds.org/). If the diagnosis was made preoperatively, these possible unsafe drugs could not have been used and, as a result, the patient could not have experienced the refractory VFs in the ICU.

The congenital LQTS is often overlooked and difficult to recognize before the occurrence of arrhythmic events, especially in patients with congenital heart diseases, as in our case. However, preoperative diagnosis of this syndrome is essential as it may help physicians to avoid arrhythmic factors [1, 2, 12]. Diagnostic difficulty is due to the fact that patients with LQTS and ASDs occasionally show a RBBB preoperatively or after surgical correction of tetralogy of Fallot, and this ECG abnormality can mask the prolonged QT time [1]. It has also been reported that QT interval is prolonged after surgical correction of ASD in 42% of patients due to increased coronary flow after the correction [13]. Thus, physicians should carefully evaluate patients’ family medical history, especially when they present RBBB and a strong family history of arrhythmias. It is important to note that Schwartz and Moss score can help medical practitioners to diagnose the LQTS, even in the absence of genetic screening for LQTS [14, 15].

## Conclusions

This case highlights the importance of taking a thorough family history in patients due to undergo open heart surgeries. Furthermore, we should pay attention to other ECG findings in patients with CRBBB, which can mask prolonged QT intervals. If preoperative diagnosis of the LQTS is made, we may be able to avoid factors triggering ventricular arrhythmias and prevent arrhythmic events after open heart surgeries.

## Abbreviations

ASD: Atrial septal defect
CPB: Cardiopulmonary bypass
CRBBB: Complete right bundle branch block
DC: Direct current
ECG: Electrocardiogram
RBBB: Right bundle branch block
TdP: Torsades de Pointes
Vf: Ventricular fibrillation
